# Impact of Extraction Method on the Detection of Quality Biomarkers in Normal vs. DFD Meat

**DOI:** 10.3390/foods10051097

**Published:** 2021-05-15

**Authors:** Laura González-Blanco, Yolanda Diñeiro, Andrea Díaz-Luis, Ana Coto-Montes, Mamen Oliván, Verónica Sierra

**Affiliations:** 1 Área de Sistemas de Producción Animal, Servicio Regional de Investigación y Desarrollo Agroalimentario (SERIDA), Ctra. AS-267, PK 19, 33300 Villaviciosa, Asturias, Spain; lgblanco@serida.org (L.G.-B.); ydineiro@serida.org (Y.D.); mcolivan@serida.org (M.O.); 2 Instituto de Investigación Sanitaria del Principado de Asturias (ISPA), Av. del Hospital Universitario, s/n, 33011 Oviedo, Asturias, Spain; acoto@uniovi.es; 3 Department of Molecular Biology, Faculty of Medicine, University of Cantabria, Av. Herrera Oria, s/n, 39011 Santander, Cantabria, Spain; ANDYLUIS@hotmail.com; 4 Department of Morphology and Cell Biology, Faculty of Medicine, University of Oviedo, Av. Julián Clavería, 6, 33006 Oviedo, Asturias, Spain

**Keywords:** cellular compartments, protein extractability, sarcoplasmic proteins, myofibrillar proteins, meat quality biomarkers, DFD meat, oxidative stress, proteomics

## Abstract

The objective of this work was to demonstrate how the extraction method affects the reliability of biomarker detection and how this detection depends on the biomarker location within the cell compartment. Different extraction methods were used to study the sarcoplasmic and myofibrillar fractions of the *Longissimus thoracis et lumborum* muscle of young bulls of the Asturiana de los Valles breed in two quality grades, standard (Control) or dark, firm, and dry (DFD) meat. Protein extractability and the expression of some of the main meat quality biomarkers—oxidative status (lipoperoxidation (LPO) and catalase activity (CAT)), proteome (SDS-PAGE electrophoretic pattern), and cell stress protein (Hsp70)—were analyzed. In the sarcoplasmic fraction, buffers containing Triton X-100 showed significantly higher protein extractability, LPO, and higher intensity of high-molecular-weight protein bands, whereas the TES buffer was more sensitive to distinguishing differences in the protein pattern between the Control and DFD meat. In the myofibrillar fraction, samples extracted with the lysis buffer showed significantly higher protein extractability, whereas samples extracted with the non-denaturing buffer showed higher results for LPO, CAT, and Hsp70, and higher-intensity bands in the electrophoretic pattern. These findings highlight the need for the careful selection of the extraction method used to analyze the different biomarkers considering their cellular location to adapt the extractive process.

## 1. Introduction

Variations in meat quality depend on the specific changes that occur at the muscle cellular structure and metabolism levels, which rely on metabolic pathways triggered during the *post-mortem* conversion of muscle into meat. Changes in the protein profile of the muscle tissue can be key to understanding these processes; as such, proteomics has become a useful tool in this field [1].

Muscle is more complex than other tissues, as the subcellular architecture of skeletal muscle is different from that of mononucleated cells [2]. Therefore, the extraction of the meat proteome is influenced by the interaction of multiple factors such as the extraction method, the protein solubility, the protein location, and the *post-mortem* changes that occur during the transformation of muscle into meat [3]. To address this complexity meat scientists commonly divide the whole proteome in two fractions, sarcoplasmic and myofibrillar, which require different extraction methods due to their different extractabilities and water solubilities.

Sarcoplasmic proteins represent the 30%–35% of the total protein content of skeletal muscle and are mainly composed of metabolic proteins located in the sarcoplasm of the muscle fibers that are soluble in water or in low-ionic-strength solutions (<0.15 M). The myofibrillar proteins account for about 50% of total proteins and are mainly composed of contractile proteins that, because of their high molecular masses, structure, and being highly interconnected [4], require the use of denaturing solutions containing urea, thiourea, reducing agents (dithiothreitol (DTT) and beta-mercaptoethanol), detergents (sodium dodecyl sulfate (SDS)), and salts for their extraction and solubilization [5,6,7]. However, Chen et al. [5] reported the use of water or low-ionic-strength media for the extraction and solubilization of myofibrillar proteins from skeletal muscle.

Considering the above, we hypothesized that the analysis of biomarkers of the conversion of muscle into meat and the ultimate meat quality may be significantly affected by the muscle extraction method. Extraction conditions, such as buffer pH, ionic strength, type of salt, extraction volume, and homogenization, influence muscle protein extractability [8,9,10]. Furthermore, some of the extraction factors (reagents, pH, and ionic strength) may not be compatible with some of the analytical procedures used to determine the presence and/or abundance of the most common biomarkers. The structure of the muscle cells results in some portions of the sarcoplasm remaining between the myofibrils, complicating their protein extraction and, therefore, the analysis of some of these biomarkers. Finally, the same extractive method may perform differently depending on whether the muscle shows a compact or deteriorated structure.

To the best of our knowledge, no previous study has determined the effect of the extraction method on the reliability of the determination of the main meat quality biomarkers in different muscle cell fractions. Therefore, the objective of this work was to identify the optimal methodology to be used for the extraction and detection of the main families of meat quality biomarkers such as those related to oxidative status, metabolic and structural proteins, and cell stress. We aimed to compare the reliability of protein extraction for a meat of standard-quality grade (Control) with that for a type of defective meat (dark, firm, and dry (DFD)), which exhibits alterations in the *post-mortem* muscle metabolism that produce a dark color and poor processing characteristics, such as higher water-holding capacity, unstructured texture, and higher spoilage [11,12,13].

## 2. Materials and Methods

### 2.1. Animals

A total of 80 young bulls from the autochthonous beef breed Asturiana de los Valles (AV) were included in this work. This breed is the second-most important in the Spanish market of protected geographical indication (PGI) fresh meat, both in production and economic value. Animals were slaughtered at 14–18 months of age, according to the commercial local market and PGI requirements, in two different slaughter batches (of 42 and 38 animals, respectively) with a one-week interval. Carcasses were transferred to a cold room at 3 °C within 2 h after slaughter. At 24 h *post-mortem*, the pH (pH24) was measured at the 13th, 10th, and 6th rib level of the *Longissimus thoracis et lumborum* (LTL) muscle of the left-half carcass using a penetration electrode coupled with a temperature probe (InLab Solids Go-ISM, Mettler-Toledo S.A.E., Barcelona, Spain). The average of the triplicate measurements was used to categorize the carcasses into two groups: Control (pH24 ≤ 6.2) and DFD (pH24 > 6.2). The pH24 threshold was set to 6.2 to ensure that the samples considered DFD were unambiguous [14]. DFD samples accounted for 9% of the total carcasses sampled. For each DFD carcass detected (*n* = 7), a carcass from the same farm, diet, transport, and weight but with a normal pH24 (5.4 to 5.5) was selected for the Control (*n* = 7) group.

### 2.2. Muscle Sample Collection

Muscle samples (20 g) were taken from the LTL at the 13th rib level at 24 h *post-mortem* for analysis of protein extractability and different biomarkers (oxidative status, sarcoplasmic and myofibrillar proteins, and stress protein). These samples were immediately snap-frozen in liquid nitrogen and stored at −80 °C until analysis.

At 24 h *post-mortem*, the LTL muscle was removed between the 6th and the 13th ribs and transported to the laboratory where it was divided into 2.5-cm steaks for the determination of the meat quality traits. The first steak was used for instrumental color and drip loss determination. The second steak was cut under sterile conditions and divided into three portions for subsequent microbiological analysis (mesophilic and *Enterobacteriaceae* total viable counts) at 3, 7, and 14 days *post-mortem*. The following three steaks were used for meat toughness measurement using the Warner–Bratzler shear force test at 3, 7, and 14 days *post-mortem*. Finally, the last steak was divided into three portions for subsequent proteomic analysis at 3, 7, and 14 days. The steaks intended for aging were vacuum-packed in 20 μm polyamide/70 μm polyethylene bags and aged in darkness under refrigerated conditions (4 ± 1 °C). After the corresponding aging period, the steaks were frozen and stored at −20 °C (−80 °C in the case of proteomics) for subsequent analysis.

### 2.3. Meat Quality Trait Measurements

Meat color was recorded on three 10 mm diameter spots on the exposed cut surface of the LTL muscle at the 7th rib level at 24 h *post-mortem*. Indicators of lightness (L*), redness (a*), and yellowness (b*) were taken after 60 min of blooming using a Minolta CM-2300d spectrophotometer, with a D65 illuminant, and a 10° standard observer in the CIE space (Konica Minolta Inc., Madrid, Spain), and the average value of the three determinations was used [15].

Meat drip loss (percent exudates) was determined by duplicates on 50 g of fresh samples taken 24 h *post-mortem* and placed in a container (Meat juice collector, Sarstedt, Germany) at 4 °C, according to the method of Honikel [16].

Meat toughness was measured on cooked meat using the Warner−Bratzler (WB) shear test as described by Diaz et al. [17]. Results are expressed as the mean WB shear force maximum load (kg) for each steak.

For microbiological analyses, meat samples were processed according to ISO 7218 (International Organization for Standardization, 2007). Firstly, each vacuum-packed portion of meat was opened (after 3, 7, and 14 days aging), a portion of 10 g was aseptically taken, and 90 mL of sterile buffered peptone water (PW, Oxoid, Basingstoke, UK) was added. The mixture was homogenized in a stomacher (IUL instruments, Barcelona, Spain) for 2 min. For microbial counts, the appropriate decimal dilutions of the samples were prepared and placed onto the corresponding medium Petri dishes. Total mesophilic aerobic microorganism counts were determined on plate count agar (PCA; Oxoid, Basingstoke, U.K.), incubated at 30 °C for 72 h; *Enterobacteriaceae* were determined on violet red bile glucose (VRBG; Oxoid, Basingstoke, UK), incubated at 37 °C for 24 h. After incubation, microbial counts were performed as described in ISO 7218:2007.

### 2.4. Muscle Extraction Methods

Figure S1 shows the flowchart of the extraction procedure for both sarcoplasmic and myofibrillar protein fractions from muscle.

#### 2.4.1. Sarcoplasmic Protein Extraction

For each sample, eight different sarcoplasmic extraction methods, resulting from different combinations of four extraction buffers and two different centrifugation steps, were tested.

The four extraction buffers used were:

1. TES buffer (TES): 10 mM Tris (pH 7.6), 1 mM EDTA (pH 8.0), 0.25 M sucrose, and 0.6% protease inhibitor cocktail (P8340, Sigma-Aldrich Co., St. Louis, MO, USA) [18].
2. Sodium buffer (Na): 50 mM sodium phosphate buffer (pH 7.5) and 0.6% protease inhibitor cocktail (P8340, Sigma-Aldrich Co., St. Louis, MO, USA) [19].
3. Sodium with Triton buffer (Na + T): 50 mM sodium phosphate buffer (pH 7.5), 0.1% Triton X-100, and 0.6% protease inhibitor cocktail (P8340, Sigma-Aldrich Co., St. Louis, MO, USA) [20].
4. Potassium with Triton buffer (K + T): 10 mM potassium phosphate buffer (pH 7.4), 50 mM NaCl, 0.1% Triton X-100, and 0.6% protease inhibitor cocktail (P8340, Sigma-Aldrich Co., St. Louis, MO, USA) [21].

The homogenization of extracts was followed by two different speed centrifugation methods:

(a) 1000× *g*, 6 min at 4 °C;
(b) 20,000× *g*, 20 min at 4 °C.

For each meat sample and extraction method, 0.5 g of muscle was homogenized in 4 mL of the corresponding extraction buffer using a Polytron PT1200 E (Kinematica Inc., Luzern, Switzerland) two times for 15 s at maximum speed. The supernatants of the seven individuals of each sample group (Control and DFD) were collected and pooled (one pool for Control and one for DFD), aliquoted, and stored at −80 °C.

#### 2.4.2. Myofibrillar Protein Extraction

For each sample, two different myofibrillar extraction methods were tested, using denaturing or non-denaturing solutions.

1. The denaturing extraction was performed on the sample residue after the extraction of sarcoplasmic proteins with the TES buffer and 20 min centrifugation at 20,000× *g* and 4 °C, as proposed by Bjarnadottir et al. [22]. The resulting pellet was homogenized into 4 mL of lysis buffer (10 mM Tris-HCl (pH 7.6), 7 M urea, 2 M thiourea, 2% CHAPS, and 10 mM DTT) with the polytron 2 × 15 s at 20,000 rpm. Subsequently, this solution was stirred for 1 h in a Multi Reax stirrer (Heidolph Instruments, Schwabach, Germany) and was centrifuged at 20,000 rpm for 20 min at 4 °C. The supernatant containing the myofibrillar proteins was collected and filtered through a nylon filter (5 mm), aliquoted, and stored at −80 °C.
2. The non-denaturing myofibrillar extraction was based on the method reported by Hashimoto et al. [23], with the following modifications: 0.5 g of muscle samples were homogenized in 4 mL of non-denaturing extraction buffer (30 mM of sodium phosphate buffer (pH 7)) and 0.6% protease inhibitor cocktail (Sigma-Aldrich Co., St. Louis, MO, USA) using a Polytron PT1200 E (Kinematica Inc., Luzern, Switzerland) two times for 15 s at maximum speed. The homogenates obtained were centrifuged at 8000× *g* for 20 min at 4 °C. The recovered pellet was resuspended in 4 mL of KCl phosphate buffer ((pH 7.5); 0.45 M KCl, 15.6 mM Na_2_PO_4_, and 3.5 mM KH_2_PO_4_) and vortexed. Subsequently, this solution was stirred for 30 min in a Multi Reax stirrer (Heidolph Instruments, Schwabach, Germany). The mixture was centrifuged twice at 5000× *g* for 15 min at 4 °C. After the centrifugation, the supernatant containing the myofibrillar proteins was recovered, aliquoted, and stored at −80 °C.

From now on, the eight different sarcoplasmic extracts are referred to as: TES 1000, TES 20,000, Na 1000, Na 20,000, Na + T 1000, Na + T 20,000, K + T 1000, and K + T 20,000, and the two myofibrillar extracts are referred to as “lysis” for the denaturing extraction and “ND” for the non-denaturing extraction.

### 2.5. Protein Extractability

The solubility of muscle proteins is the amount of protein remaining in a solution of defined characteristics after the application of a specific centrifugal force for a determined duration. The terms solubility and extractability are frequently interchanged, assuming that once the protein is solubilized, it can be readily extracted from muscle fibers or myofibrils into a solution [24]. The protein content of the different extracts was measured by the Bradford method [25].

### 2.6. Oxidative Stress

The oxidative status of the muscle tissue was assessed by the measurement of lipid oxidative damage (lipoperoxidation (LPO)) and catalase activity (CAT). LPO was analyzed by measuring the reactive aldehyde malondialdehyde (MDA) and 4-hydroxy-2-(E)-nonenal (4-HNE) using the LPO assay kit from Calbiochem (No.437634, San Diego, CA, USA) [26], which measures lipid hydroperoxides directly using redox reactions with ferrous ions, and the results are expressed as nmol MDA + 4-HNE/g protein.

Catalase activity (CAT; EC 1.11.1.6) was analyzed according to the method developed by Lubinsky and Bewley [27] using hydrogen peroxidase (H_2_O_2_) as the substrate. The results are expressed as μmol H_2_O_2_/min mg protein.

### 2.7. Sarcoplasmic and Myofibrillar Subproteome Analysis

The separation of proteins obtained with the different extraction buffers was performed using SDS-PAGE gels as described by Díaz et al. [17], with minor modifications. Sarcoplasmic (15 μg of protein) and myofibrillar (30 μg of protein) muscle extracts were denatured with sample buffer (65.8 mM Tris/HCl (pH 6.8), 2% SDS, 21% glycerol, 5% beta-mercaptoethanol, and 0.026% bromophenol blue) and boiled at 100 °C for 5 min. Samples were loaded into 1-mm dual vertical slab gels (Mini-PROTEAN^®^ Tetra Cell, Bio-Rad Laboratories Inc., Hercules, CA, USA) and run for 2.50 h (sarcoplasmic extracts) or 2.20 h (myofibrillar extracts) at 150 V for one-dimensional electrophoresis (1D-SDS-PAGE). The resolving gel contained 12% and the stacking gel 4% of acrylamide/bis (30% acrylamide), 10% (*w/v*) SDS, 1.5 M Tris/HCl (pH 8.8), 0.5 M Tris/HCl (pH 6.8), 10% (*w/v*) ammonium persulphate, and 0.1% TEMED. Prestained molecular weight standards (Precision Plus Protein™ All Blue Standards, Bio-Rad Laboratories Inc., Hercules, CA, USA) were run on each gel to determine the protein band molecular weights. Gels were stained (50% methanol, 10% acetic acid, and QC Colloidal Coomassie from Bio-Rad) and afterward de-stained with distilled water. Three gels per sample were performed.

Stained-gel images were captured using the UMAX ImageScanner (Amersham Biosciences, Buckinghamshire, UK). SDS-PAGE densitometry analysis and band quantification were performed as described by Díaz et al. [17].

### 2.8. Stress Protein: Hsp70

Stressors, such as high temperature, hypoxia, ischemia, and oxidation, can induce the synthesis of stress proteins like the heat shock proteins (Hsps) to protect cellular proteins against denaturation [27]. Among the best-known and most-investigated Hsps is the Hsp70 family. Hsp70 is abundantly induced in the response to cellular stress in muscles [28] and it was proposed to be a key biomarker of the process of conversion of muscle into meat and, therefore, of the ultimate meat quality, as it can simultaneously indicate the tenderness, color, and WHC of meat [29], which are some of the quality traits that are more affected in DFD meat. Therefore, the expression of Hsp70-1A/B was measured by Western blotting. The homogenized tissue (90 µg protein per sample) was mixed with Laemmli sample buffer (65.8 mM Tris/HCl (pH 6.8)), 2% SDS, 21% glycerol, 5% beta-mercaptoethanol, and 0.026% bromophenol blue) and denatured by boiling at 100 °C for 5 min. The extracts were fractionated using SDS-PAGE at 200 V, and then proteins were transferred to polyvinylidene fluoride membranes (Immobilon TM-P; Millipore Corp., Burlington, MA, USA) at 350 mA. Once the membranes were blocked at 4 °C overnight with 10% (*w*/*v*) bovine seroalbumin (BSA) dissolved in Tris-buffered saline (TBS) (50 mM Tris-HCl and 150 mM NaCl, (pH 7.5)), they were incubated at 4 °C overnight with the primary antibody anti-Hsp70 (A5A) (ab2787, Abcam, Cambridge, U.K.), which detects Hsp70-1A/B (UniProtKB: P0DMV8/P0DMV9). The antibody was pre-diluted in TBS buffer containing 5% (*w*/*v*) BSA. After three washes in TBS-T (50 mM Tris-HCl (pH 7.5), 150 mM NaCl, and 0.05% Tween-20), the membranes were incubated with the corresponding horseradish-peroxidase-conjugated secondary antibody (Cell Signaling, Danvers, MA, USA) and diluted in TBS buffer with BSA 2% (*w*/*v*) for 1 h at room temperature. After three washes in TBS-T, the immunoconjugates were detected using a chemiluminescent horseradish peroxidase substrate (WBKLS0500, Millipore Corp., Darmstadt, Germany) according to the manufacturer’s instructions. The Image Studio Lite 5.2.5 program (LI-COR Biosciences, Lincoln, NE, USA) allowed us to quantify the optical density of the bands. The densitometry results are expressed as semi-quantitative optical density (in arbitrary units) of blot bands, normalized to Ponceau bands as the loading control. Three replicates per sample were performed.

### 2.9. Statistical Analysis

The normality of variables was tested using the Kolmogorov–Smirnoff test. The effect of sample type (Control vs. DFD) on the different quality traits was analyzed by a *t*-test of independent samples. For variables measured at different *post-mortem* times (WBSF, microbiological loads), the effect of aging time (with animal as the random factor) was tested. For the rest of the variables included in the study, the effect of extraction method E (eight different extraction methods for sarcoplasmic extracts and two for myofibrillar extracts) and the effect of sample type T (Control vs. DFD), and their interaction (E × T) were analyzed by ANOVA using the general linear model procedure in SPSS v. 22.0 (SPSS Inc., Chicago, IL, USA). Once the interaction between E and T was established, the effects of the extraction method and the type of sample were tested independently. When significant, differences between extraction methods were analyzed by means of Tukey’s post hoc test, and the Games–Howell test when variances were not homogeneous.

## 3. Results and Discussion

### 3.1. Meat Quality Traits

As expected, DFD meat had a higher pH24 (*p* < 0.001), darker color (L *, *p* < 0.001), was less red (a *, *p* < 0.01) and less yellow (b *; *p* < 0.001), and had a higher growth of mesophilic (*p* < 0.001) and *Enterobacteriaceae* (*p* < 0.005) microorganisms at 14 days of aging (Table 1).

Previous studies comparing meat from three different Spanish autochthonous breeds reported similar results for color traits with significantly higher values of L*, a*, and b* in high-pH (>6) meat from Asturiana de los Valles and Rubia Gallega [30]. Poleti et al. [31] reported lower values of the color parameters in high-pH meat when comparing beef from Nellore cattle classified into two different pH groups: high (≥6.0) and normal (<5.8). It is known that due to the high pH, DFD meat is more prone to microbial spoilage than normal-pH meat [13]; accordingly, we found a faster reduction in the shelf life of the DFD meat at 14 days *post-mortem*. In agreement with our results, García-Torres et al. [30] found similar results with higher mesophlic loads in Rubia Gallega and Asturiana de los Valles breeds at 7 and 14 days of aging. No significant differences were found for WBSF between the Control and DFD samples in this study; however, an anomalous tenderization process was observed in DFD meat as meat toughness did not significantly decrease with aging.

Samples with a high pH24 (>6.2) in the present study were darker and their microbial spoilage was higher, so they were therefore of defective quality compared with those with a lower pH. These differences in quality traits may reflect differences at the muscle cell level (structure and metabolism), which have to be considered to understand the results obtained in this study.

### 3.2. Protein Extractability

**Figure 1:**
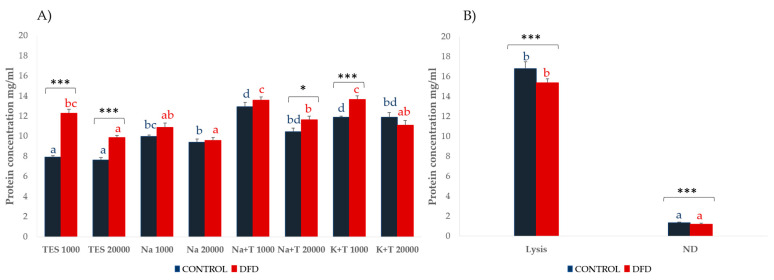
Protein content (mean ± SEM) of the (**A**) sarcoplasmic and (**B**) myofibrillar fractions from Control (blue) and DFD (red) meat samples and the different extraction methods. Charts with different letters (blue for Control and red for DFD) were significantly different between extraction methods at *p* < 0.05. Asterisks indicate significant differences between Control and DFD samples within the same extraction method. * *p* < 0.05; *** *p* < 0.001. TES 1000: TES buffer and 1000× *g*, 6 min; TES 20,000: TES buffer and 20,000× *g*, 20 min; Na 1000: sodium phosphate buffer and 1000× *g*, 6 min; Na 20,000: sodium phosphate buffer and 20,000× *g*, 20 min; Na + T 1000: sodium phosphate buffer with Triton X-100 and 1000× *g*, 6 min; Na + T 20,000: sodium phosphate buffer with Triton-X100 and 20,000× *g*, 20 min; K + T 1000: potassium phosphate buffer with Triton X-100 and 1000× *g*, 6 min; K + T 20,000: potassium phosphate buffer with Triton X-100 and 20,000× *g*, 20 min; Lysis: denaturing extraction with lysis buffer; ND: non-denaturing extraction.

The protein contents of the sarcoplasmic and myofibrillar fractions obtained by the different extraction methods tested are shown in Figure 1.

In the sarcoplasmic fraction, higher extractability was obtained with buffers containing Triton X-100 (Na + T and K + T), which is a type of non-ionic detergent used for cell lysis, that is, for the disruption of cell membranes and the consequent release of intracellular materials that breaks protein–lipid and lipid–lipid associations, and generally does not denature proteins. The higher protein content in these extracts could be explained by Triton X-100 helping to solubilize most membrane proteins in their native and active form, retaining their protein interactors. In the sarcoplasmic fraction, the centrifugation speed only affected the protein solubility of some DFD extracts (TES, Na + T, and K + T), being significantly higher (*p* < 0.05) at 1000× *g*, whereas no significant differences were found for the Control samples. This could be related to a higher disintegration of the muscle structure in DFD meat, which resulted in the higher extraction capability of sarcoplasmic proteins retained within the sarcoplasm portions embedded between the myofibrils.

In the myofibrillar fraction, the lysis buffer showed significantly higher protein extractability than ND for both Control and DFD samples. Lysis buffer contains some agents such as urea, thiourea, CHAPS, and DTT, which may have been responsible for these extractability differences [32]. Urea is a chaotropic agent that denatures proteins by disrupting noncovalent and ionic links between amino acids [33], whereas thiourea improves the solubilization of hydrophobic membrane proteins [34]; therefore, their combination is used to extract proteins that are otherwise insoluble. CHAPS prevents hydrophobic interaction and DTT aids in the solubilization of complex mixtures by reduction of disulfide bonds, avoiding protein aggregation or precipitation [35]. The combination of these components increases solubilization and proteins extractability [36,37].

### 3.3. Oxidative Stress

**Figure 2:**
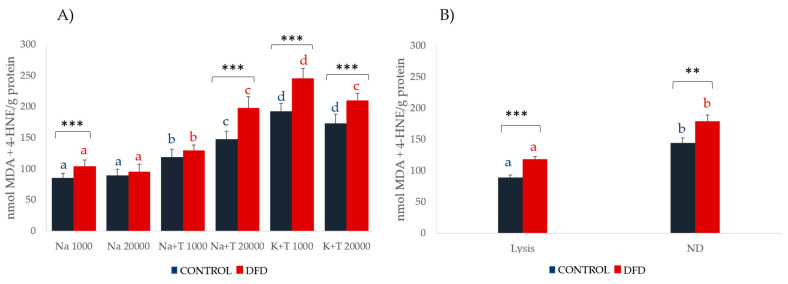
Lipoperoxidation (mean ± SEM) of (**A**) sarcoplasmic and (**B**) myofibrillar fractions from Control (blue) and DFD (red) meat samples. Charts with different letters (blue for and red for DFD) were significantly different between extraction methods at *p* < 0.05. Asterisks indicate significant differences between the Control and DFD samples within the same extraction procedure. ** *p* < 0.01; *** *p* < 0.001. Na 1000: sodium phosphate buffer and 1000*× g*, 6 min; Na 20,000: sodium phosphate buffer and 20,000*× g*, 20 min; Na + T 1000: sodium phosphate buffer with Triton X-100 and 1000*× g*, 6 min; Na + T 20,000: sodium phosphate buffer with Triton-X100 and 20,000*× g*, 20 min; K + T 1000: potassium phosphate buffer with Triton X-100 and 1000*× g*, 6 min; K + T 20,000: potassium phosphate buffer with Triton X-100 and 20,000*× g*, 20 min; Lysis: denaturing extraction with lysis buffer; ND: non-denaturing extraction.

**Figure 3:**
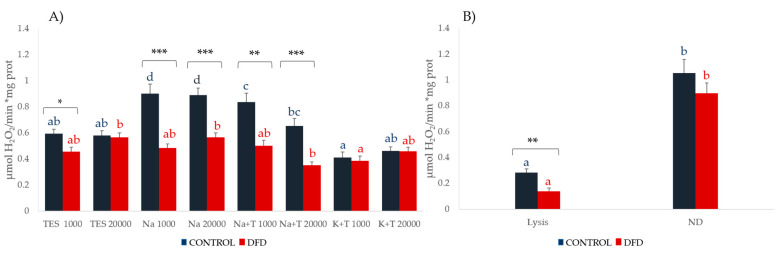
Catalase activity (mean ± SEM) of the (**A**) sarcoplasmic and (**B**) myofibrillar fractions from Control (blue) and DFD (red) meat samples. Charts with different letters (blue for Control and red for DFD) were significantly different between extraction methods at *p* < 0.05. Asterisks indicate significant differences between the Control and DFD samples within the same extraction procedure. * *p* < 0.05; ** *p* < 0.01; *** *p* < 0.001. TES 1000: TES buffer and 1000*× g*, 6 min; TES 20,000: TES buffer and 20,000*× g*, 20 min; Na 1000: sodium phosphate buffer and 1000*× g*; 6 min; Na 20,000: sodium phosphate buffer and 20,000*× g*, 20 min; Na + T 1000: sodium phosphate buffer with Triton X-100 and 1000*× g*, 6 min; Na + T 20,000: sodium phosphate buffer with Triton-X100 and 20,000*× g*, 20 min; K + T 1000: potassium phosphate buffer with Triton X-100 and 1000*× g*, 6 min; K + T 20,000: potassium phosphate buffer with Triton X-100 and 20,000*× g*, 20 min; Lysis: denaturing extraction with lysis buffer; ND: non-denaturing extraction.

Figure 2 and Figure 3 show the results for LPO and CAT in both the sarcoplasmic and myofibrillar fractions. The TES buffer was incompatible with some reagents present in the LPO assay kit (probably EDTA) and produced unstable results, so the results of the TES extracts were not considered for this assay.

DFD samples showed higher LPO values (Figure 2) in all extracts and in both cellular fractions. This could be related to a higher pre-slaughter stress situation, which increases the oxidative damage of lipids in the cells of the animals that finally produced DFD carcasses. Within the sarcoplasmic fraction, the K + T 1000 extraction method showed higher LPO values (*p* < 0.001) in both the Control and DFD samples, whereas in the myofibrillar fraction, the ND buffer showed higher LPO values (*p* < 0.001).

CAT activity was higher in the Control samples in both the sarcoplasmic and myofibrillar extracts, which seems to be related to a higher antioxidant defense in the muscle of standard-quality meat obtained in the absence of pre-slaughter stress. In the sarcoplasmic extracts, higher CAT activity was found in the samples extracted with Na buffer independent of the centrifugation speed. However, its determination in extracts containing Triton X-100 was difficult due to the non-ionic detergents interfering with ultra-violet (UV) spectrophotometry, thus producing unstable results.

In the myofibrillar fraction, CAT activity was significantly higher in the extracts obtained by the non-denaturing method (*p* < 0.05).

### 3.4. Sarcoplasmic and Myofibrillar Subproteome

**Figure 4:**
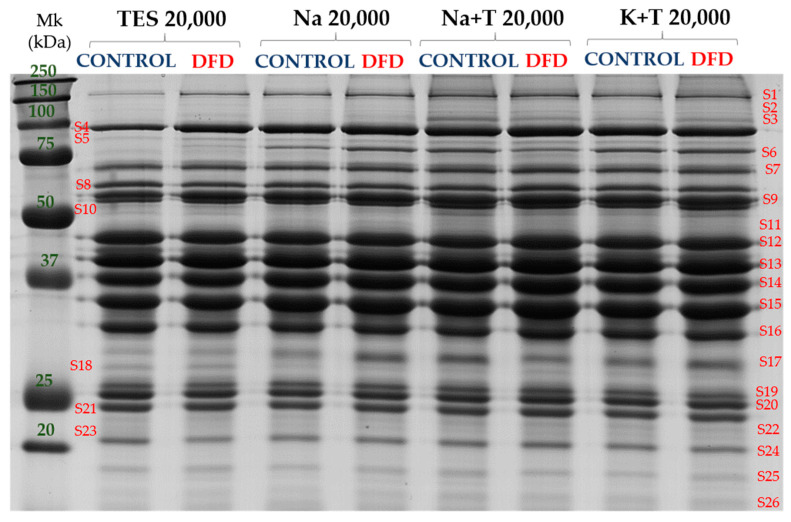
The 1D-SDS-PAGE gel image of sarcoplasmic subproteome from the Control and DFD meat samples extracted with different buffers (TES, Na, Na + T, and K + T) at 20,000*× g*. Mk: prestained molecular weight marker (All Blue prestained, Biorad). Band names are denoted by S (sarcoplasmic protein) followed by a number.

SDS-PAGE gels allowed for the separation of 26 protein bands (ranging from 15 to 200 kDa) from the muscle sarcoplasmic subproteome, as shown in Figure 4, which shows the protein pattern obtained with the different extraction methods at the maximum centrifugation speed tested (20,000*× g*) for both types of meat samples (Control and DFD).

The complete details of the results (means ± SEM) for the significant sarcoplasmic bands obtained with the different extraction methods and type of samples are provided in Table S1. The analysis of the main factors studied (extraction method, type of sample, and their interaction) showed a significant interaction for four bands (S1, S4, S18, and S21). Once these bands were discarded, the differences between extraction methods were analyzed including all the samples regardless of being Control or DFD. Table 2 shows a total of 13 sarcoplasmic bands with significant differences in band intensity between extraction methods.

Bands of higher molecular weight (over 50 kDa) showed higher intensities when using the Na + T 20,000 method, whereas protein bands under 50 kDa showed higher intensities with the TES buffer. The majority of the sarcoplasmic bands separated by 1D SDS-PAGE fell into the <50 kDa molecular weight range; the TES buffer seems to be a better option for studying the sarcoplasmic subproteome.

When studying the effect of sample type, the extractions made with the TES buffer showed more protein bands (S1, S3, S4, S5, S9, S10, S15, S16, S18, S19, S20, and S21) with significant differences (*p* < 0.05) between the Control and DFD samples (Table S2), which reinforces the conclusion that this method of extraction is the most suitable for the electrophoretic analysis of the sarcoplasmic fraction of the muscle tissue. When skeletal muscle is homogenized in a sucrose medium, as in the case of TES buffer, it forms a gelatinous consistency that inhibits the disruption of the myofibrils; therefore, the differences found between the Control and DFD extracts reflect the differences in the *post-mortem* evolution of the myofibril disruption (faster in the defective and unstructured DFD meat), thus reporting an essential information of the differences in biomarker patterns between both meat types.

**Figure 5:**
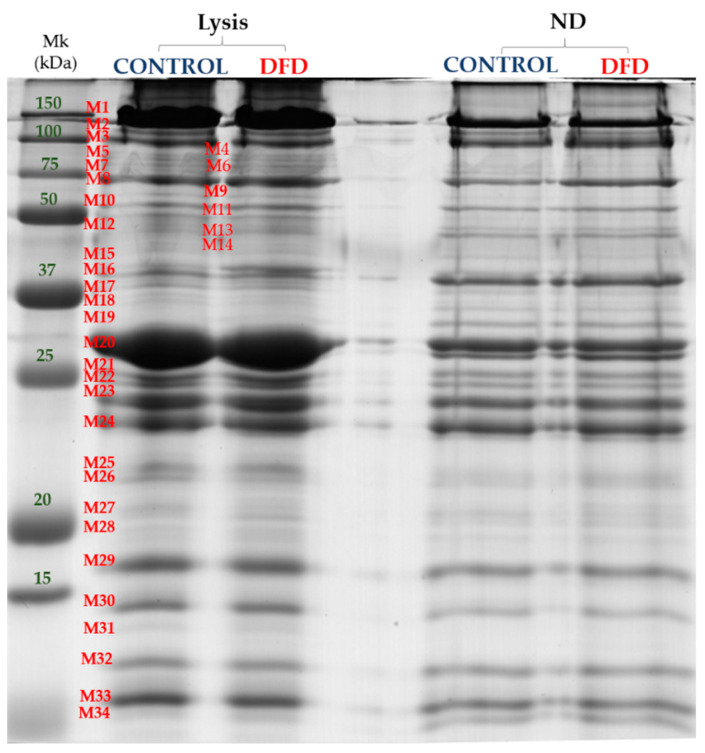
The 1D-SDS-PAGE gel image of myofibrillar subproteome from Control and DFD meat samples extracted with different buffers: lysis and non-denaturing buffer (ND). Mk: prestained molecular weight marker (All Blue prestained, Biorad). Band names are denoted by M (myofibrillar protein) followed by a number.

In the myofibrillar fraction, despite the large differences (*p* < 0.001) in protein extractability, both extracts provided a similar and adequate separation of 34 well-defined protein bands in the range of molecular weights from 15 to 250 kDa (Figure 5).

The complete details of the results (means ± SEM) for significant myofibrillar bands are provided in Table S3. The analysis of the main factors (extraction method, type of sample, and their interaction) showed a significant interaction of extraction method and sample type for three bands (M16, M17, and M26). Once these bands were discarded, differences between extraction methods were compared including all the samples regardless of being Control or DFD. Table 3 shows a total of 14 myofibrillar bands with significant differences in band intensity between the extraction methods.

Overall, 10 bands (M2, M3, M6, M18, M19, M24, M27, M32, and M34) showed higher intensity in the non-denaturing extracts, which indicated that the ND method, even despite its lower protein extractability, adequately separated well-defined myofibrillar protein bands by SDS-PAGE. Lysis buffer resulted in major band intensity for M11, M20, M23, M30, and M31 bands.

The effect of sample type was also analyzed for each buffer independently. In the lysis extracts, seven bands (M10, M16, M17, M26, M31, M32, and M34) showed significant differences between the Control and DFD meat, whereas only two bands (M26 and M31) showed significant differences in the ND extracts (Table S4). Therefore, it seems that the lysis buffer was more sensitive to changes in the muscle structure, probably due to the denaturing conditions increasing the extraction of proteins of a low molecular weight [32], leading to differences between samples of different muscle structure and compactness.

Considering the results for the sarcoplasmic and myofibrillar subproteome, it seems that some proteins were easily extracted with most of the buffers, whereas others remained linked to cellular organelles and membranes or in the sarcoplasm portions embedded within the myofibrils, which complicated their extraction. The intensity of these effects depends on the evolution of the muscle dismantlement in the process of conversion of muscle into meat and the resulting meat quality grade.

### 3.5. Stress Protein: Hsp70

Proteomic studies have reported the differential expression of Hsp70 in meat with variable quality traits [22,38]. Several studies have correlated meat quality with Hsp70 under stress situations [39,40,41].

**Figure 6:**
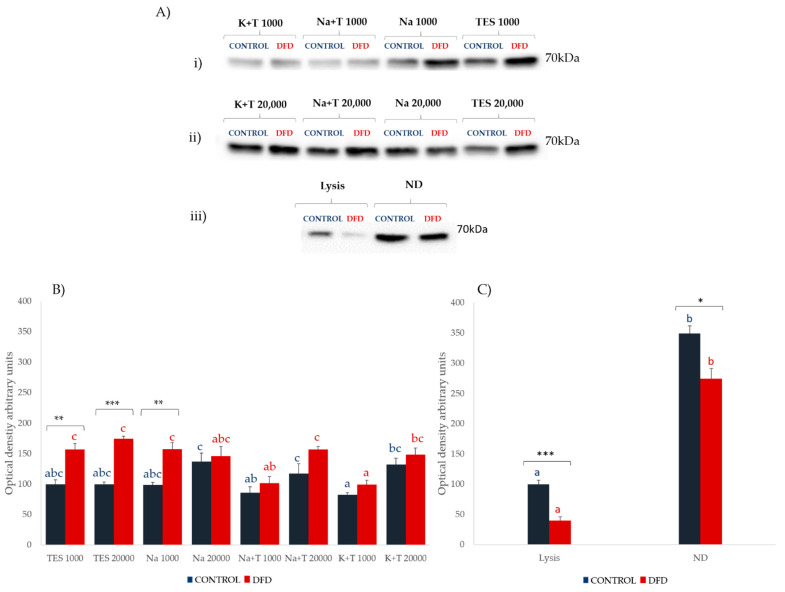
Hsp70 Western blotting results. (**A**) Representative immunoblot analyses of Hsp70 protein expression of sarcoplasmic extracts at (i) 1000*× g* and (ii) 20,000*× g*, and (iii) Hsp70 expression of myofibrillar extracts. Ponceau staining was used as a loading control. (**B**) Expression of Hsp70 (mean ± SEM from three independent experiments) of sarcoplasmic extracts (left) and (**C**) myofibrillar extracts (right) from Control (blue) and DFD (red) meat samples. Charts with different letters (blue for Control and red for DFD) were significantly different between extraction methods at *p* < 0.05. Asterisks indicate significant differences between the Control and DFD samples within the same extraction procedure. * *p* < 0.05; ** *p* < 0.01; *** *p* < 0.001. TES 1000: TES buffer and 1000*× g*, 6 min; TES 20,000: TES buffer and 20,000*× g*, 20 min; Na 1000: sodium phosphate buffer and 1000*× g*, 6 min; Na 20,000: sodium phosphate buffer and 20,000*× g*, 20 min; Na + T 1000: sodium phosphate buffer with Triton X-100 and 1000*× g*, 6 min; Na + T 20,000: sodium phosphate buffer with Triton-X100 and 20,000*× g*, 20 min; K + T 1000: potassium phosphate buffer with Triton X-100 and 1000*× g*, 6 min; K + T 20,000: potassium phosphate buffer with Triton X-100 and 20,000*× g*, 20 min; Lysis: denaturing extraction with lysis buffer; ND: non-denaturing extraction.

Our results (Figure 6) showed clear differences in Hsp70 expression between the sarcoplasmic and myofibrillar fractions. For the sarcoplasmic fraction, higher Hsp70 expression was found in DFD samples, with these differences being significant for the TES 1000, TES 20,000, and Na 1000 extraction methods. However, the sarcoplasmic fraction of the Control samples showed higher Hsp70 expression when extracted with sodium buffers and higher centrifugal speed (Na 20,000 and Na + T 20,000).

For the myofibrillar fraction, higher Hsp70 expression was found for ND extracts, but contrary to what was observed for the sarcoplasmic fraction, the Control samples showed higher Hsp70 expression independent of the extractive method used (lysis and ND). This higher expression in Control samples from the myofibrillar extracts may have been due to the protective role that Hsp70 played against muscle dismantlement in the early *post-mortem* stages. Previous studies showed that the majority of Hsp70 is readily diffusible within the cytoplasm in non-stressed muscle fibers; after stress, Hsp70 primarily binds to and stabilizes the structure and function of cell membranes [42]. Furthermore, under stress situations such as during muscle-damaging exercise, Hsp70 translocates and accumulates to the cytoskeletal and myofibrillar proteins [43]. Xing et al. [44] found that Hsp70 was present in the cytoplasm and on the surface membranes of cells from the *Pectoralis major* muscle in normal-quality chicken meat following stress. Hsp70 was present on the surface membranes and extracellular matrix but was barely visible in the cytoplasm of the PSE-like samples, that is, low-quality meat due to stress. This diffusion capacity of Hsp70 may explain the differences found in this work between extracts. Under the stressful situation produced from slaughter, Hsp70 may translocate to the myofibrillar fraction in an attempt of stabilize the muscle structure, which would explain its increased expression in the myofibrillar fraction of the Control samples. However, in the DFD meat, more Hsp70 is easily removed and extracted from the sarcoplasmic fraction, showing the movement of Hsp70 from the inner myofibrillar compartment to the sarcoplasm due to the faster dismantlement of the muscle.

## 4. Conclusions

Within the sarcoplasmic fraction, buffers containing Triton X-100 led to a higher protein extractability, LPO detection, and determination of proteins with high molecular weight. However, TES buffer was more sensitive for the detection of Hsp70 expression and the electrophoretic bands of lower molecular weight, showing increased ability to discriminate between the meat samples with different metabolisms and the degree of cell dismantlement (Control vs. DFD). In the myofibrillar fraction, the non-denaturing buffer reported higher LPO, CAT activity, and Hsp70 expression, and showed higher intensity bands in the electrophoretic pattern; however, the lysis buffer increased protein extractability and its electrophoretic pattern was more sensitive to differences between the Control and DFD samples.

These findings highlight the need to select the most appropriate extraction method for each biomarker family and muscle structure type, and the need to consider different cell fractions and the movements of proteins between cytoskeletal and myofibrillar structures, for an accurate and reliable study of the process of conversion of the muscle into meat.

## Figures and Tables

**Table 1:** Effect of quality grade (Control vs. DFD) on meat quality traits (mean ± standard deviation).

Variable	Time *post-mortem*	Control (*n* = 7)	DFD (*n* = 7)	Sig.
pH Drip loss (%) L*	24 h	5.48 ± 0.05	6.49 ± 0.27	***
	48 h	1.19 ± 0.64	1.06 ± 0.31	NS
	48 h	34.35 ± 2.53	27.71 ± 2.34	***
a* b*	48 h	9.84 ± 2.82	5.83 ± 0.97	**
	48 h	11.87 ± 2.45	6.14 ± 2.38	***
Meat toughness (WBSF, kg)	3 days	7.15 ± 1.74b	6.63 ± 2.50	NS
	7 days	6.02 ± 1.31ab	5.56 ± 1.85	NS
	14 days	4.97 ± 1.01a	5.33 ±1.35	NS
Mesophilic (log UFC/kg)	3 days	3.73 ± 0.37a	3.72 ± 1.06a	NS
	7 days	4.31 ± 0.84a	4.42 ± 1.71a	NS
	14 days	6.05 ± 0.37b	7.22 ± 0.64b	***
*Enterobacteriaceae* (log UFC/kg)	3 days	1.26 ± 1.23	1.53 ± 1.32a	NS
	7 days	1.45 ± 1.43	2.13 ± 1.64a	NS
	14 days	3.08 ± 1.59	4.91 ± 0.83b	*

For variables measured at different times *post-mortem* (meat toughness, mesophilic, and *Enterobacteriaceae*), means in the same column followed by different letters differ statistically. DFD: dark, firm and dry; Sig.: Significance; NS: not significant; * *p* < 0.05; ** *p* < 0.01; *** *p* < 0.001.

**Table 2:** Effect of the extraction method on the sarcoplasmic subproteome bands intensity (optical density in arbitrary units).

Sarcoplasmic Bands (MWe ^1^)	TES 1000	TES 20,000	Na 1000	Na 20,000	Na + T 1000	Na + T 20,000	K + T 1000	K + T 20,000	SEM	Sig.
S2 (137.9 kDa)	0.188	0.211	0.246	0.262	0.431	0.446	0.42	0.316	0.061	**
S3 (115.8 kDa)	0.305a	0.249a	0.406ab	0.344a	0.753bc	0.911c	0.602abc	0.421ab	0.084	***
S6 (81.31 kDa)	0.55a	0.572a	1.556b	1.256b	1.442b	1.559b	1.705b	1.482b	0.146	***
S10 (53.60 kDa)	0.895a	0.992ab	0.998ab	1.031ab	1.336b	1.325b	1.211ab	1.197ab	0.087	**
S11 (50.70 kDa)	1.264abc	1.107a	1.244abc	1.223ab	1.438abcd	1.68d	1.603bcd	1.611cd	0.086	***
S12 (45.55 kDa)	8.244ab	8.814b	8.101ab	7.837ab	7.536ab	7.049a	7.474ab	6.874a	0.351	**
S13 (40.72 kDa)	10.805b	10.448ab	10.159ab	10.154ab	9.258a	9.34ab	8.513a	8.98a	0.427	**
S14 (37.6 kDa)	8.775ab	8.707ab	9.287b	9.16ab	8.524ab	8.413ab	8.18a	8.345ab	0.242	*
S15 (34.74 kDa)	10.859bcd	10.457abc	11.59d	11.376cd	10.74abcd	9.77a	10.341abc	9.857ab	0.241	***
S16 (32.14 kDa)	8.128b	8.079b	6.672a	6.878a	6.53a	6.136a	6.446a	6.38a	0.211	***
S17 (29.74 kDa)	1.576a	1.826a	2.399c	2.835cd	2.649cd	2.677cd	3.28d	3.179d	0.109	***
S19 (26.68 kDa)	2.889b	2.89b	2.258a	2.546ab	2.419ab	2.585ab	2.22a	2.521ab	0.128	***
S20 (25.76 kDa)	4.162b	4.103b	3.635ab	3.661ab	3.425a	3.329a	3.482a	3.483a	0.133	***

Means within a row followed by different letters were significantly different at * p < 0.05; ** *p <* 0.01; *** *p <* 0.001. ^1^ MWe is the experimental molecular weight (kDa); SEM: standard error of the mean; Sig.: significance.

**Table 3:** Effect of extraction method on the myofibrillar subproteome band intensity (optical density in arbitrary units).

Myofibrillar Bands(MWe ^1^)	Lysis	ND	SEM	Sig.
M2 (170.8 kDa)	1.667	2.464	0.142	**
M3 (143.58 kDa)	3.139	5.893	0.417	***
M6 (110.53 kDa)	0.719	1.066	0.096	***
M11 (74.77 kDa)	0.896	0.500	0.063	**
M18 (49.7 kDa)	0.698	1.245	0.113	***
M19 (47.58 kDa)	0.899	1.717	0.104	**
M20 (41.07 kDa)	14.276	8.959	1.033	**
M23 (34.80 kDa)	5.503	4.660	0.242	*
M24 (32.76 kDa)	4.874	7.404	0.303	***
M27 (26.31 kDa)	1.466	2.128	0.085	***
M30 (19.46 kDa)	3.033	2.388	0.128	***
M31 (18.40 kDa)	0.693	0.406	0.051	***
M32 (17.09 kDa)	2.254	3.100	0.117	***
M34 (14.94 kDa)	0.817	2.314	0.123	***

Means within a row were significantly different at * *p* < 0.05; ** *p* < 0.01; *** *p* < 0.001. ^1^: Mwe: the experimental molecular weight (kDa); Sig.: significance; Lysis: denaturing extraction with lysis buffer; ND: non-denaturing extraction.

## Data Availability

Data are available upon request.

## Supplementary Materials

The following are available online at https://www.mdpi.com/article/10.3390/foods10051097/s1, Figure S1. Flowchart of the different extraction methods for sarcoplasmic and myofibrillar proteins from beef muscle.1: TES buffer; 2: soidum phosphate buffer; 3: Naa+T: sodium phosphate buffer with triton X-100; K+T: potassium phosphate buffer with Triton X-100; ND: non-denaturing extraction. Table S1: Effect of extraction method (TES 1000, TES 20,000, Na 1000, Na 20,000, Na+T 1000, Na+T 20,000, K+T 1000 and K+T 20,000), type of sample (CONTROL vs. DFD) and their interaction on sarcoplasmic subproteome bands’ intensity (optical density in arbitrary units). Table S2: The *p*-values for the effect of sample type (CONTROL vs. DFD) on the sarcoplasmic subproteome bands intensitiy (optical density in arbitrary units) obtained with the different extration methods. Table S3: Effect of extraction method (Lysis and Non-denaturant), type of sample (CONTROL vs. DFD) and their interaction on myofibrillar subproteome bands’ intensity (optical density in arbitrary units). Table S4: Effect of meat type (CONTROL vs. DFD) within each extraction method (Lysis vs. Non-denaturant) on myofibrillar subproteome bands’ intensity (optical density in arbitray units).
