# Supplementary material for: Impact of Extraction Method on the Detection of Quality Biomarkers in Normal vs. DFD Meat
Source: Foods. 2021 May 15;10(5):1097. doi: 10.3390/foods10051097 (PMC8156798; doi:10.3390/foods10051097)
Supplement: Supplementary file 1 [file foods-10-01097-s001.zip › foods-1193786-SI.pdf]

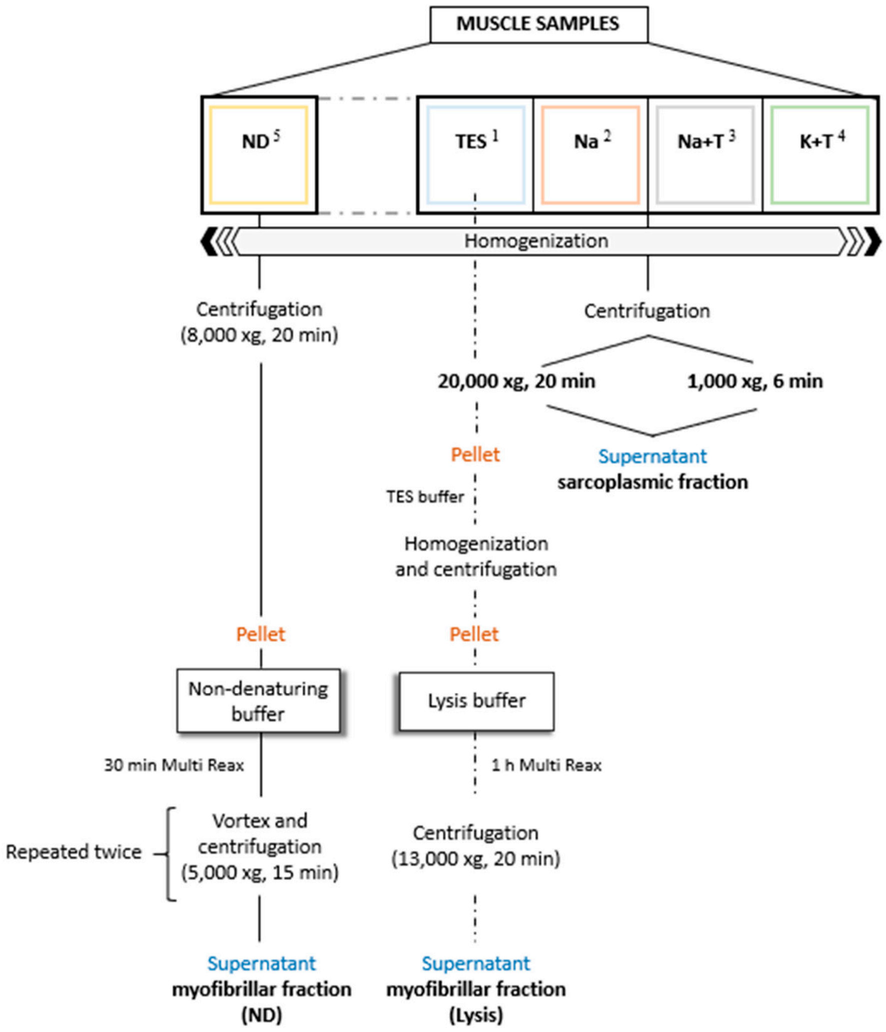

Figure S1. Flowchart of the different extraction methods for sarcoplasmic and myofibrillar proteins from beef muscle. [1]:TES: TES buffer; [2]: Na: sodium phosphate buffer; [3]:Na+T: sodium phosphate buffer with Triton X-100; [4]:K+T: potassium phosphate buffer with Triton X-100 ; [5]:ND: non-denaturing extraction.

Table S1. Effect of extraction method (TES 1000, TES 20,000, Na 1000, Na 20,000, Na+T 1000, Na+T 20,000, K+T 1000 and K+T 20,000), type of sample (CONTROL *vs.* DFD) and their interaction on sarcoplasmic subproteome bands' intensity (optical density in arbitrary units).

Sarcoplasmic bands (MWe[1])	TES 1000		TES 20,000		Na 1000		Na 20,000		Na+T 1000		Na+T 20,000		K+T 1000		K+T 20,000			Significance		
	CONTROL	DFD	CONTROL	DFD	CONTROL	DFD	CONTROL	DFD	CONTROL	DFD	CONTROL	DFD	CONTROL	DFD	CONTROL	DFD	SEM	E	T	E x T
S1 (171.1 kDa)	0.322	1.374	0.488	1.459	1.551	2.027	1.675	1.963	2.310	1.876	1.857	2.764	2.326	1.399	2.500	1.67	0.213	***	NS	**
S2 (137.9 kDa)	0.127	0.249	0.165	0.256	0.224	0.268	0.214	0.309	0.43	0.431	0.372	0.519	0.415	0.425	0.278	0.352	0.61	*	NS	NS
S3 (115.8 kDa)	0.163	0.446	0.155	0.344	0.335	0.477	0.298	0.388	0.876	0.63	0.824	0.998	0.528	0.676	0.322	0.518	0.084	***	*	NS
S4 (95.3 kDa)	3.156	5.6	3.350	5.771	5.059	5.241	5.771	5.192	4.526	4.046	5.136	5.318	4.722	3.865	4.857	3.622	0.32	*	NS	***
S5 (87.9 kDa)	0.382	0.593	0.597	1.150	0.647	0.99	1.323	1.631	1.109	0.807	1.958	1.585	1.021	0.974	1.754	1.719	0.382	NS	NS	NS
S6 (81.31 kDa)	0.443	0.657	0.553	0.591	1.190	1.920	1.144	1.368	1.455	1.428	1.321	1.797	1.783	1.625	1.466	1.497	0.146	***	NS	NS
S8 (62.48 kDa)	2.931	2.637	2.961	2.745	2.93	2.74	2.958	2.519	2.942	2.847	3.029	2.84	2.924	2.272	2.643	2.502	0.123	NS	**	NS
S10 (53.60 kDa)	0.756	1.034	0.923	1.061	0.985	1.011	1.012	1.049	1.225	1.448	1.25	1.399	1.224	1.198	1.091	1.303	0.087	**	*	NS
S11 (50.70 kDa)	1.364	1.163	1.126	1.087	1.255	1.232	1.209	1.235	1.483	1.392	1.619	1.741	1.786	1.420	1.531	1.691	0.086	***	NS	NS
S12 (45.55 kDa)	8.409	8.078	9.198	8.428	7.687	8.515	8.381	7.293	7.677	7.394	6.614	7.485	7.559	7.388	6.89	6.858	0.351	**	NS	NS
S13 (40.72 kDa)	10.617	10.99	10.34	10.55	9.696	10.62	10.49	9.813	9.189	9.326	9.332	9.348	8.792	8.235	9.029	8.932	0.427	**	NS	NS
S14 (37.6 kDa)	8.947	8.603	8.983	8.432	9.474	9.099	9.635	8.685	8.365	8.682	8.334	8.492	8.418	7.942	8.499	8.191	0.242	*	NS	NS
S15 (34.74 kDa)	11.212	10.51	10.86	10.06	11.84	11.34	11.55	11.21	10.52	10.96	9.488	10.05	10.46	10.23	9.728	9.985	0.241	***	NS	NS
S16 (32.14 kDa)	8.765	7.491	8.764	7.394	6.728	6.615	7.100	6.656	6.425	6.634	6.097	6.175	6.738	6.154	6.499	6.261	0.211	***	**	NS
S17 (29.74 kDa)	1.650	1.502	1.738	1.913	2.151	2.646	2.418	3.251	2.495	2.804	2.430	2.924	2.914	3.646	2.909	3.448	0.109	***	***	NS
S18 (28.41 kDa)	1.521	1.352	1.627	1.479	0.976	1.393	1.070	1.315	1.067	1.506	1.072	1.229	1.351	1.52	1.337	1.49	0.073	***	**	*
S19 (26.68 kDa)	3.163	2.615	3.162	2.618	2.223	2.292	2.672	2.419	2.421	2.416	2.595	2.575	2.189	2.249	2.436	2.606	0.128	**	NS	NS
S20 (25.76 kDa)	4.395	3.929	4.275	3.936	3.588	3.681	3.605	3.717	3.556	3.294	3.471	3.187	3.458	3.506	3.623	3.342	0.133	***	NS	NS
S21 (24.63 kDa)	4.227	3.712	4.058	3.778	3.401	3.467	3.392	3.381	3.312	3.340	3.209	3.221	3.164	2.963	3.092	3.036	0.057	***	**	*

[1:] MWe is the experimental molecular weight (kDa); E: Extractive method; T: Type of sample (CONTROL vs DFD); SEM: standard error of the mean; NS: Not significant; * $p < 0.05$; ** $p < 0.01$; *** $p < 0.001$. TES 1000: TES buffer and 1000× g, 6 min; TES 20,000: TES buffer and 20,000× g, 20 min; Na 1000: sodium phosphate buffer and 1000× g, 6 min; Na 20,000: sodium phosphate buffer and 20,000× g, 20 min; Na+T 1000: sodium phosphate buffer with Triton X-100 and 1000× g, 6 min; Na+T 20,000: sodium phosphate buffer with Triton-X100 and 20,000× g, 20 min; K+T 1000: potassium phosphate buffer with Triton X-100 and 1000× g, 6 min; K+T 20,000: potassium phosphate buffer with Triton X-100 and 20,000× g; 20 min

Table S2. The *p*-values for the effect of sample type (CONTROL *vs.* DFD) on the sarcoplasmic subproteome bands intensitiy (optical density in arbitrary units) obtained with the different extration methods.

**Sarcoplasmic bands** (MWe[1])	**TES 1000**	**TES 20,000**	**Na 1000**	**Na 20,000**	**Na+T 1000**	**Na+T 20,000**	**K+T 1000**	**K+T 20,000**
S1 (171.1 kDa)	**0.001*** **	**0.002** **	**0.04** **	0.372	0.444	0.221	0.201	0.05
S3 (115.8 kDa)	**0.007** **	0.085	0.065	0.379	0.258	0.629	0.436	0.136
S4 (95.3 kDa)	**0.001*** **	**0.001*** **	0.735	0.425	0.546	0.809	0.096	0.257
S5 (87.9 kDa)	**0.028***	**0.047***	0.523	0.722	0.583	0.739	0.944	0.975
S8 (62.48 kDa)	0.064	0.438	0.383	0.207	0.782	0.496	**0.021***	0.509
S9 (57.6 kDa)	**0.003** **	**0.027***	0.768	0.488	0.664	0.833	0.621	0.891
S10 (53.60 kDa)	**0.045***	0.413	0.901	0.845	0.208	0.596	0.807	0.272
S15 (34.74 kDa)	**0.004** **	**0.04***	0.069	0.511	0.42	0.529	0.513	0.687
S16 (32.14 kDa)	**0.011***	**0.028***	0.686	0.369	0.637	0.891	0.195	0.567
S17 (29.74 kDa)	0.363	0.185	**0.027***	**0.000*** **	0.402	0.151	**0.05***	**0.000*** **
S18 (28.41 kDa)	**0.03***	0.385	**0.019***	0.23	0.019*	0.315	0.414	0.272
S19 (26.68 kDa)	**0.029***	0.057	0.833	0.193	0.982	0.962	0.702	0.573
S20 (25.76 kDa)	**0.018***	0.148	0.496	0.362	0.392	0.541	0.876	0.447
S21 (24.63 kDa)	**0.011***	0.099	0.575	0.889	0.813	0.939	0.082	0.45

p-values in bold are significant at * $p < 0.05$; ** $p < 0.01$; *** $p < 0.001$. [1]: Mwe is the experimental molecular weight (kDa). TES 1000: TES buffer and 1000× g, 6 min; TES 20,000: TES buffer and 20,000× g, 20 min; Na 1000: sodium phosphate buffer and 1000× g, 6 min; Na 20,000: sodium phosphate buffer and 20,000× g, 20 min; Na+T 1000: sodium phosphate buffer with Triton X-100 and 1000× g, 6 min; Na+T 20,000: sodium phosphate buffer with Triton-X100 and 20,000× g, 20 min; K+T 1000: potassium phosphate buffer with Triton X-100 and 1000× g, 6 min; K+T 20,000: potassium phosphate buffer with Triton X-100 and 20,000× g; 20 min

Table S3. Effect of extraction method (Lysis and Non-denaturant), type of sample (CONTROL *vs.* DFD) and their interaction on myofibrillar subproteome bands' intensity (optical density in arbitrary units).

Myofibrillar bands (MWe[1])	Lysis		ND			Significance		
	CONTROL	DFD	CONTROL	DFD	SEM	E	T	E x T
M2 (170.8 kDa)	1.617	1.717	2.338	2.590	0.245	**	NS	NS
M3 (143.58 kDa)	3.212	3.066	5.432	6.354	0.644	***	NS	NS
M6 (110.53 kDa)	0.855	0.583	1.104	1.029	0.112	*	NS	NS
M11 (74.77 kDa)	0.935	0.856	0.590	0.410	0.09	***	NS	NS
M16 (55.70 kDa)	2.982	2.080	3.920	4.849	0.385	***	NS	*
M17 (52.15 kDa)	1.418	0.792	1.244	1.140	0.086	NS	**	*
M18 (49.7 kDa)	0.773	0.623	1.220	1.270	0.111	**	NS	NS
M19 (47.58 kDa))	1.013	0.785	1.677	1.757	0.102	***	NS	NS
M20 (41.07 kDa)	14.011	14.542	9.890	8.029	2.035	**	NS	NS
M23 (34.80 kDa)	5.506	5.500	4.327	4.993	0.305	*	NS	NS
M24 (32.76 kDa)	4.651	5.098	6.953	7.855	0.564	***	NS	NS
M25 (29.16 kDa)	1.841	1.568	1.946	1.092	0.153	NS	*	NS
M26 (28.48 kDa)	0.987	1.234	1.254	0.792	0.097	NS	NS	*
M27 (26.31 kDa)	1.514	1.418	2.182	2.074	0.065	***	NS	NS
M30 (19.46 kDa)	2.958	3.109	2.337	2.439	0.178	**	NS	NS
M31 (18.40 kDa)	0.756	0.630	0.526	0.286	0.067	***	**	NS
M32 (17.09 kDa)	2.081	2.426	2.900	3.299	0.139	***	*	NS
M34 (14.94 kDa)	0.906	0.729	2.478	2.151	0.122	***	NS	NS

[1:] Mwe is the experimental molecular weight (kDa); E: Extraction method; T: Type of sample (CONTROL *vs.* DFD); SEM: standard error of the mean; NS: Not significant; * $p < 0.05$; ** $p < 0.01$; *** $p < 0.001$. Lysis: denaturing extraction with lysis buffer; ND: non-denaturing extraction.

Table S4. Effect of meat type (CONTROL *vs.* DFD) within each extraction method (Lysis *vs* Non-denaturant) on myofibrillar subproteome bands' intensity (optical density in arbitray units).

	Lysis			ND		
**Myofibrillar bands** (MWe[1])	**CONTROL**	**DFD**	**Sig.**	**CONTROL**	**DFD**	**Sig.**
M10 (79.89 kDa)	1.593	1.142	*	1.687	1.465	NS
M16 (55.70 kDa)	2.982	2.080	*	3.920	4.849	NS
M17 (52.15 kDa)	1.418	0.792	*	1.244	1.140	NS
M26 (28.48 kDa)	0.987	1.234	*	1.254	0.792	*
M31 (18.40 kDa)	0.756	0.630	*	0.526	0.286	*
M32 (17.09 kDa)	2.081	2.426	*	2.900	3.299	NS
M34 (14.94 kDa)	0.906	0.729	***	2.478	2.151	NS

[1]: Mwe is the experimental molecular weight (kDa); Sig.: Significance. Means within a row and extractive method were significantly different at: * $p < 0.05$; *** $p < 0.001$. NS: not significant. . Lysis: denaturing extraction with lysis buffer; ND: non-denaturing extraction.
